# Percutaneous Coronary Intervention Outcomes Among Patients With Alcoholic Cardiomyopathy: An Analysis of the National Inpatient Sample

**DOI:** 10.7759/cureus.29490

**Published:** 2022-09-23

**Authors:** Endurance O Evbayekha, Bede N Nriagu, Gabriel Alugba, Okelue E Okobi, Ovie Okorare, Geraldine Nsofor, Ihuoma A Ngoladi, Chinelo Igweike, Maureen G Boms, Nkemputaife P Onyechi, Aisha M Abubakar, Eniola Olamilehin, Akinbanji R Afolabi, Uduak A Udo, Evidence E Ohikhuai

**Affiliations:** 1 Internal Medicine, St. Luke's Hospital, St. Louis, USA; 2 Internal Medicine, New York Medical College, Metropolitan Hospital Center, New York, USA; 3 Internal Medicine, Delta State University, Abraka, NGA; 4 Family Medicine, Lakeside Medical Center, Belle Glade, USA; 5 Internal Medicine, Milton Keynes University Hospital, Milton Keynes, GBR; 6 Family Medicine, Independent Researcher, Calgary, CAN; 7 Preventive Medicine, RBH Medical Center, Richmond, USA; 8 Clinical Research, University of Alabama at Birmingham, Birmingham, USA; 9 Internal Medicine, University Hospitals Cleveland Medical Center, Cleveland, USA; 10 Family Medicine, University of Maiduguri, Maiduguri, NGA; 11 Internal Medicine, St. George's University School of Medicine, St. George’s, GRD; 12 Internal Medicine, Emory University, Atlanta, USA; 13 Radiology, Imaging for Women, Kansas City, USA; 14 Public Health, Jackson State University School of Public Health, Jackson, USA

**Keywords:** percutaneous coronary intervention, inpatient outcomes, average length of hospital stay, alcohol dependence, nationwide inpatient sample (nis), outcome measure, primary percutaneous coronary intervention (pci), alcoholic cardiomyopathy

## Abstract

Introduction

High consumption of alcohol has an enormous toll on the health status of individuals. A direct affectation of cardiac integrity concerns cardiologists, primary care physicians, and the healthcare system because this increases the disease burden. Alcoholic cardiomyopathy (ACM) results from the enormous consumption of alcohol over a long period of time. The prevalence varies between regions and sex and ranges between 4% and 40%. Viewing the entire spectrum of cardiomyopathies, ACM makes up about 4% of all cardiomyopathies. However, it causes dilated-type cardiomyopathy and is the second most common cause of dilated cardiomyopathy. We sought to explore the outcomes of percutaneous coronary intervention (PCI) among patients with ACM.

Methods

This was a retrospective, cross-sectional study of the National Inpatient Sample (NIS) for hospital discharges in the United States between 2012 and 2014. We identified the number of patients with a primary or secondary diagnosis of ACM using the International Classification of Diseases, Ninth Revision (ICD-9) code of 4.255. Using the ICD-9 codes for PCI (00.66, 36.01, 36.02, 36.05, 36.06, 36.07, and 17.55), we identified patients diagnosed with ACM who underwent a PCI (ACPCI). The racial and sexual prevalence, hospital length of stay (LOS), mortality, cost of hospitalization, and cardiovascular outcomes (ventricular fibrillation (VF) and atrial fibrillation (AF)) were compared between patients with and without ACM who underwent a PCI.

Results

A total of 2,488,293 PCIs were performed between 2012 and 2014. Of these, there were a total of 161 admissions for ACM. About 93% (151) of the ACM PCI group were men. Ethnic distribution revealed a majority of Caucasians with 69% (98), and blacks and Asians at 13.4% (19) and 11.3% (16), respectively. The mean age was 59.8 (SD = 9). The patients with ACPCI were likely to stay longer in the hospital, with an average stay of 6.6 days (SD = 6.2) compared to patients without ACM undergoing PCI (NOACPCI) (3.7 days; SD = 5.0) (p = 0.0001). The mean cost of hospital admission for patients with ACPCI was $120,225 (SD = 101,044), while that of those without ACM who underwent PCI (NOACPCI) was $87,936 (SD = 83,947) (p = 0.0001). A higher death rate during hospitalization (3.7%) was recorded in the ACPCI category vs. 2.3% in patients without ACM who underwent PCI (p = 0.0001). Patients with ACPCI had a higher prevalence of AF (30.4%) than VF (7.5%).

Conclusion

The ACPCI group had overall poorer hospital outcomes. The majority affected were older Caucasian men with an increased prevalence of AF, higher cost of hospitalization, and longer hospital stays. Further studies are needed to explore the burden of long-term alcohol consumption on cardiovascular disease treatment outcomes.

## Introduction

Alcoholism is broadly discussed under two terms: alcohol abuse and alcohol dependence. Alcohol abuse refers to heavy alcohol intake for adequate functioning, while alcohol dependence is defined as an increased alcohol tolerance with clinical manifestations upon cessation of intake [1]. In the 2019 National Survey on Drug Use and Health (NSDUH), 85.6% of individuals above the age of 18 reported alcohol intake at least once during their lifetime, 69.5% indulged in the past year, and 54.9% reported alcohol use in the past month [2]. Alcoholic cardiomyopathy (ACM) is a cardiac disease characterized by cardiac and skeletal muscle dysfunction among patients who consume >80 g of alcohol per day for equal to or greater than five years [3]. ACM has been recognized as one of the main causes of left ventricular dysfunction in developed countries. In the United States, ACM is the leading cause of non-ischemic dilated cardiomyopathy [4]. Globally, the total mortality of ACM was 71,723 with an age-specific death rate of less than one per 100,000 persons, and the global ACM-related disability-adjusted life years were 2,441,108 with an age-standardized disability-adjusted life years rate of 29.20 per 100,000 persons in 2019 [5]. Some studies reflected the beneficial effects of minimal alcohol consumption. This observation may be concerning because the risk of developing cardiovascular diseases may be obscured by confounding factors such as lifestyle, lower tobacco smoking rates, optimal body weight, higher physical activity, and a healthy diet. There is human genetic evidence portraying a causal relationship between alcohol intake and cardiovascular diseases like hypertension and coronary artery disease that are consistently on the rise, especially with higher levels of alcohol consumption [6]. The treatment of heart failure in ACM involves medications that decrease the workload of the heart with diuretics, angiotensin-converting enzyme inhibitors, beta-blockers being the mainstay for the achievement of euvolemia and improvement in signs of decompensated heart failure, and non-pharmacological therapy like alcohol cessation, reduction of salt intake, and healthy diet [7]. There is a paucity in the use of percutaneous coronary intervention (PCI) in managing ACM. This study focused on patients who underwent PCI for ACM during hospitalization, with outcomes significant for longer length of stay (LOS), more costly hospitalization, and increased incidence of atrial fibrillation (AF).

## Materials and methods

We conducted a retrospective analysis using the weighted hospital discharge data provided by the Healthcare Cost and Utilization Project - National Inpatient Sample (NIS). We chose to focus on the dataset from 2012 to 2014 because no trend weight is needed to create a national estimate for the NIS dataset from 2012 and beyond. The NIS is a publicly available deidentified database that reflects about 8 million hospitalizations annually, representing 20% of all admissions across the United States.

The NIS is designed so that data representation of each hospitalization contains clinically relevant data such as a patient's diagnosis, current or past medical histories, and procedures. Hospitalized patients 18 years or older with a primary or secondary diagnosis of "alcoholic cardiomyopathy" were included in the study using the International Classification of Diseases, Ninth Revision, Clinical Modification (ICD-9-CM) diagnosis code 4.255. Furthermore, we identified patients using the ICD-9-CM codes for PCI (00.66, 36.01, 36.02, 36.05, 36.06, 36.07, and 17.55) and included patients who had a diagnosis of ACM and underwent PCI. The ICD-9-CM codes used in our study for ACM and PCI have previously been validated by Ram et al. (2018) and Alkhouli et al. (2020), respectively [3,8].

Study outcomes

The primary outcomes were in-hospital mortality and cardiovascular events, i.e., AF and ventricular fibrillation (VF). In contrast, secondary outcomes included LOS, the cost of hospitalization, differences in outcomes between ethnic categories, and the type of insurance of the patients. Incomplete patient records and patients transferred from the hospital were excluded from the study.

Study population

The study included patients aged 18 years or older who were admitted with a primary or secondary diagnosis of ACM, i.e., DX1-DX30; ICD-9-CM code was 4.255. This population was identified using the ICD-9-CM codes for primary PCI (00.66, 36.01, 36.02, 36.05, 36.06, 36.07, and 17.55) and patients who had a diagnosis of ACM who also underwent PCI.

Analysis

Weighted data from 2012 to 2014 were used for statistical analyses. We preferred the 2012 dataset because the NIS 2012 and beyond database was redesigned to overcome discrepancies that plague the dataset of prior years, such as the exclusion of long-term care facilities and sample stratification by nine census divisions rather than four. The 2012 and beyond NIS database was drawn from 47 states, including the state of Maryland, representing 97% of the US population [9].

We applied a descriptive statistical method for demographics and baseline characteristics of patients, which are presented as numbers with percentages. The mean ± one SD was used to express continuous data and analysis was done using the analysis of variance (ANOVA). Variables in our analysis include demographic and socioeconomic factors such as age, sex, ethnicity, median household income, and primary expected payer. We considered results with p-values of <0.05 as statistically significant. All analyses were performed using Statistical Analysis System (SAS) software version 9.4 (SAS Institute Inc., Cary, NC).

## Results

**Figure 1:**
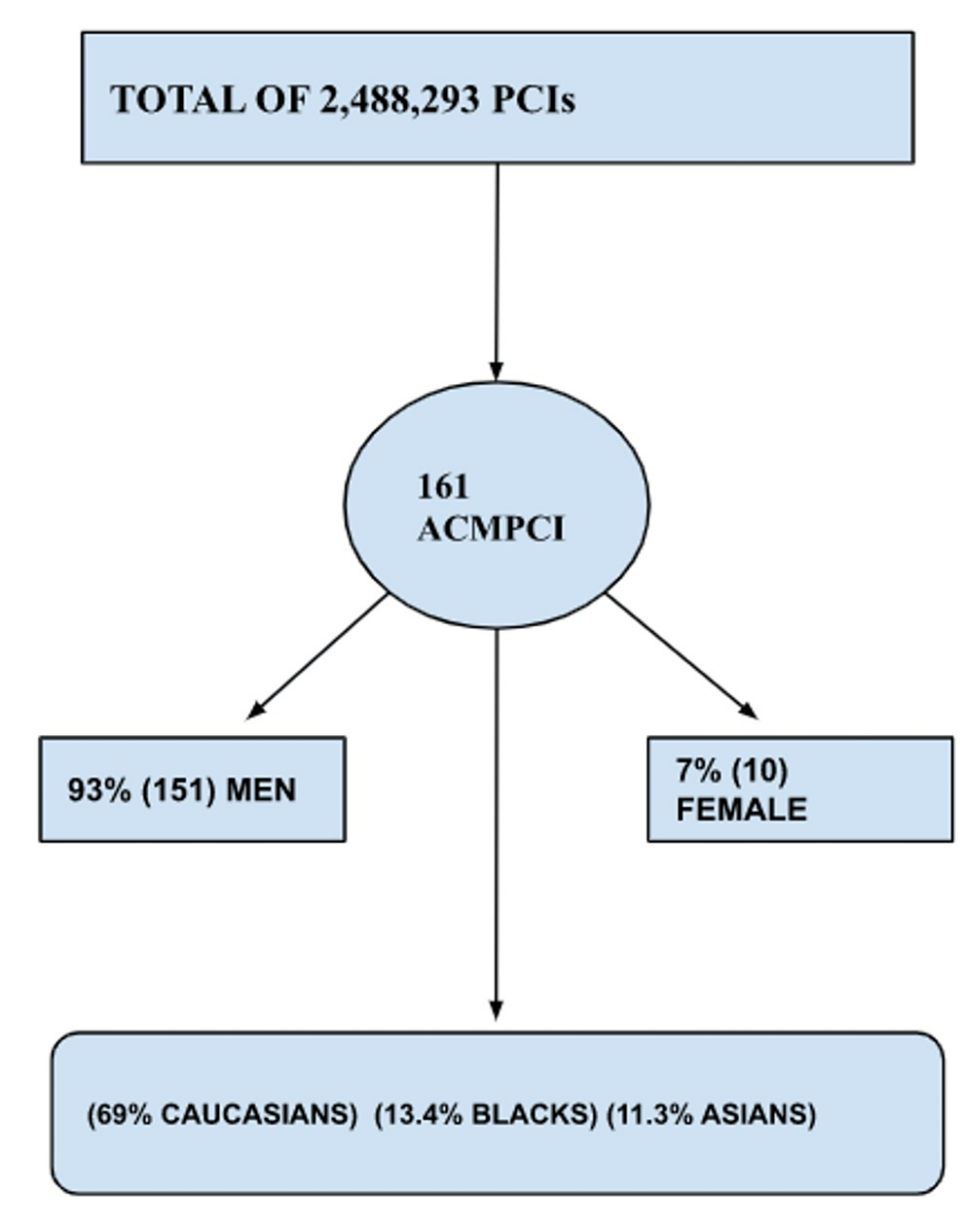
Flowchart illustration of data analysis and results PCIs = percutaneous coronary interventions; ACMPCI = alcoholic cardiomyopathy individuals who underwent PCI.

A total of 2,488,293 PCIs were performed between 2012 and 2014. Of these, there were 161 admissions for ACM, as illustrated in Figure 1. About 93% (151) of the ACM PCI group were men. Ethnic distribution revealed a majority of Caucasians with 69% (98), and blacks and Asians at 13.4% (19) and 11.3% (16), respectively. However, there was no statistical difference between prevalence in ethnic groups as p = 0.09. There was no difference in outcomes for patients admitted to the hospitals over the weekend versus weekdays (p = 0.8). The patients were further classified according to insurance type, with the predominant insurance being Medicare (70, 43.85%), private insurance (41, 25.6%), Medicaid (24, 15%), and self-pay (22, 13.7%).

The patients with ACM who underwent PCI (ACPCI) were likely to stay longer in the hospital, with an average stay of 6.6 days (SD = 6.2) compared to patients without ACM undergoing PCI (NOACPCI) (3.7 days, SD = 5.0) (p = 0.0001). The mean cost of hospital admission for patients with ACPCI was $120,225 (SD = 101,044), while that of those without ACM who underwent PCI (NOACPCI) was $87,936 (SD = 83,947) (p = 0.0001). A higher death rate during hospitalization (3.7%) was recorded in the ACPCI category vs. 2.3% in NOACPCI (p = 0.0001). Patients with ACPCI had a higher prevalence of AF (30.4%) than VF (7.5%). In contrast with the ACPCI cohort, the NOACPCI had 33.8% (3476/10434) of AF. There was no statistical significance as p = 0.4. However, there was a significant difference between the VF prevalence in ACPCI (7.5%, 12/161) and NOACPCI (1.79%, 190/10434) as p = 0.001.

**Figure 2:**
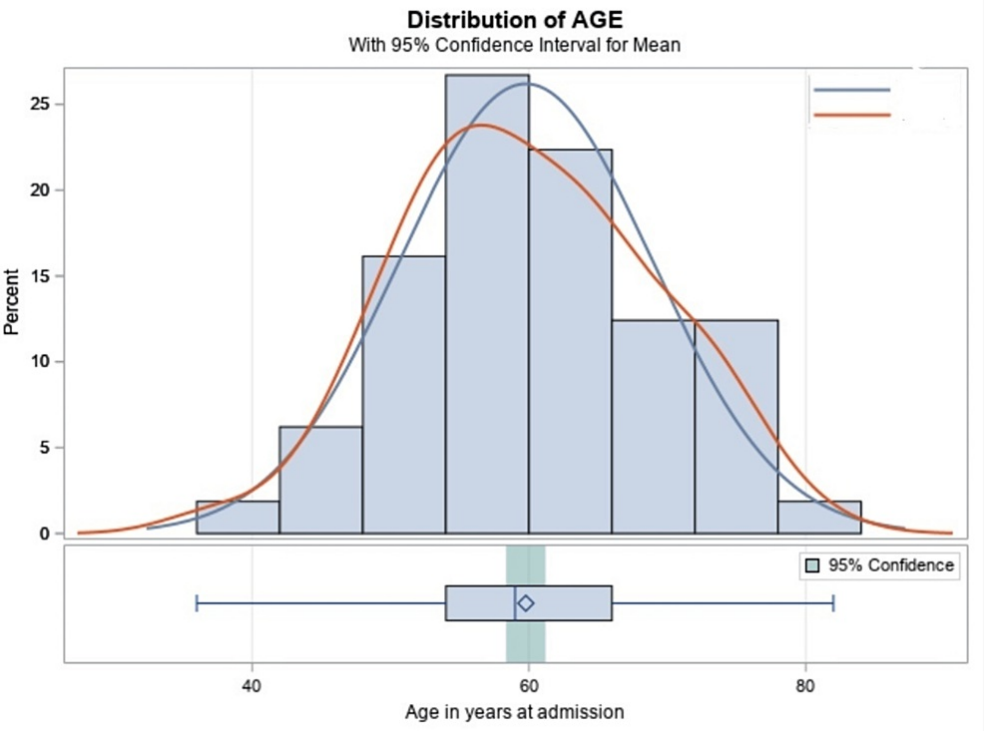
Diagram representing the mean age of patients with alcoholic cardiomyopathy undergoing PCI Red curve = patients with alcoholic cardiomyopathy undergoing PCI. Blue curve = individuals without alcoholic cardiomyopathy undergoing PCI. PCI = percutaneous coronary intervention.

Below in Figure 2, we represented the ACPCI group using the red curve. The peak represents the mean age of 59 years (SD = 9). This is much younger than their NOACPCI counterparts, represented by the blue curve with a late-onset peak, with a mean age of 65 years (SD = 10).

## Discussion

This study showed 161 patients hospitalized for ACM over the duration of two years; this low prevalence of hospital admission for ACM is in keeping with studies that were done by Sulaiman et al. [10], which showed a decreasing number of hospitalizations of patients with ACM per 100,000 of US patients (4.38 in 2005 vs. 3.62 in 2014). Ram et al. also found a decreasing trend for ACM hospitalization [3]. This could be due to the increasing awareness of the deleterious effects of alcohol and enhanced public measures to curb the rate of alcohol intake. This is evidenced by a decreasing trend in the annual per capita intake of alcohol in the United States, although there has been a plateau since 2007 [11]. However, data show increased alcohol use since 2012 compared to the early 2000s [12]. Studies have revealed that advances in healthcare utilization, social support, insurance coverage, and comorbidity burden have played a key role in the declining trend of ACM-related hospitalization [13,14].

Sulaiman et al. noted that the median length of patients hospitalized for ACM was four days [10]. Our study revealed an increase in the LOS (6.6 days) of ACM patients who had PCI compared to patients without ACM undergoing PCI (3.7 days). This could be a result of in-hospital cardiovascular complications, as patients with ACM may also have other comorbidities such as hypertension, diabetes mellitus, arrhythmias, dyslipidemia, peripheral artery disease, prior stroke, substance abuse, and chronic liver disease [15], which may affect outcomes of procedures undertaken. A study by Guzzo-Merello et al. to describe the outcome of patients treated for ACM showed that one-third of patients died or had heart transplantation, and a third remained clinically stable without improved cardiac function. A third experienced substantial cardiac recovery [16]. Our study revealed that patients with ACM who had PCI had a significantly higher mortality rate compared to patients without ACM who had PCI. This may result from progressive systolic dysfunction in some ACM patients. However, the severity of illness, comorbidities, heavy habitual alcohol intake, and coexisting nutritional deficiencies may worsen cardiac procedure outcomes in ACM patients.

In our study, we identified that the mean hospital charge for patients with ACM who had PCI was significantly higher than for those without ACM who underwent PCI. This is consistent with other studies, which showed an increasing healthcare cost for patients with ACM who had different cardiac procedures done during hospitalization [3,10]. Although the increased hospital charge for patients is multifactorial, rising healthcare costs and disease severity of presenting patients are major determinants. Overall, ACM patients have other comorbidities, which may result in increased healthcare costs due to their effects on successful procedures.

Our study showed that patients with ACM who had PCI had a higher prevalence of AF (30.4%) than VF (7.5%). These findings are consistent with that of Sulaiman et al., where AF was seen to be the most common type of arrhythmia in patients with ACM (30.7%), followed by ventricular tachycardia (8.6%) [10]. Compared to the ACPCI cohort, the NOACPCI had 33.8% (3476/10434) of AF. There was no statistical significance as p = 0.4. However, there was a statistically significant difference between the VF prevalence in ACPCI (7.5%, 12/161) to NOACPCI (1.79%, 190/10434) as p = 0.001. Ebner et al. also revealed that ACM patients had a significantly higher proportion of VF/flutter [15]. Heavy habitual intake of alcohol is a well-known risk factor for AF [17] due to its effects on the atrial excitation-contraction coupling due to the inhibition of calcium release from the sarcoplasmic reticulum and cardiac tissue fibrosis it causes. Alcohol exerts oxidative stress, protein damage, and lipid peroxidation in cardiac tissues [18,19]. Some patients with ACM are known to have myocarditis with lymphocytic infiltrates accompanied by focal necrosis [7,19]. This usually presents clinically as shortness of breath and other signs of heart failure [7,11].

The earlier onset of acute coronary syndrome in patients with ACM was also documented by Ebner et al. [15]. Our study saw a younger age group requiring PCIs at 59 years (SD = 9) while the NOACPCI cohort had a mean age of 65 years (SD = 10). This result is multifactorial, possibly due to accelerated atherosclerosis and the direct toxic effect of alcohol on cardiac myocytes. The dilated type of cardiomyopathy favors the stasis of blood in the left ventricle, resulting in embolus formation and thromboembolic phenomenon [15,16]. The dilation of the cardiomyocyte results in the inability to maintain adequate contractile function and hence poor coronary blood supply to the cardiac myocyte, resulting in mixed ischemic heart disease [15]. Further exacerbation of the heart damage from more alcohol intake may result in a profound drop in contractile properties leading to an acute coronary event [15,17].

Strengths

Our study has highlighted the impact of ACM on the prognosis of patients undergoing PCI. One of the strengths of this study is the lower likelihood of bias, as this is a retrospective analysis of records. Also, we have pulled together a reasonable cohort of ACM patients undergoing PCI for study, and there are scarce data on this cohort. Hence, we can add to the body of knowledge for prognostication in clinical practice. While the NIS dataset does not differentiate between a current and previous diagnosis, ACM is a chronic disease. Hence, it is unlikely that the patient’s ACM has resolved at the current hospital admission. This gives our study a great collection of the alcohol cardiomyopathy spectrum.

Limitations

The NIS is administrative data used by hospital administration for the purposes of billing. Hence, it is prone to overbilling, underbilling, and other human errors. It is also prone to having multiple missing datasets, which may skew the analysis. It is also important to note that a single data in the NIS corresponds to one hospitalization. Duplication can arise if a single patient is transferred between hospitals. Lastly, while we sought to study the outcomes of ACPCI, the NIS dataset cannot stratify patients into the severity of the disease.

## Conclusions

Although ACM is a spectrum of different severities, the overall outcome of these cohorts undergoing a PCI is poorer than their counterparts. The burden it directly places on the healthcare system and public health, in general, is a preventable epidemic that needs attention at the least. Our study has highlighted one of the many ways alcohol complicates treatment and hospitalization costs across all ethnic groups, sex, and age. The impact of alcohol abuse on the overall health reflects an increased disease burden and is a public health concern. More studies are required to bolster evidence further and lead guideline-directed advocacy against the alcoholic menace in our society.
